# Maladaptive personality traits are associated with burnout risk in Italian anesthesiologists and intensivists: a secondary analysis from a cross-sectional study

**DOI:** 10.1186/s44158-024-00171-5

**Published:** 2024-06-21

**Authors:** Alessandro Vittori, Emiliano Petrucci, Marco Cascella, Elena Giovanna Bignami, Alessandro Simonini, Giacomo Sollecchia, Gilberto Fiore, Alessandro Vergallo, Franco Marinangeli, Roberto Pedone

**Affiliations:** 1 Department of Anesthesia and Critical Care, ARCO ROMA, Ospedale Pediatrico Bambino Gesù IRCCS, 00165 Rome, Italy; 2 Department of Anesthesia and Intensive Care Unit, San Salvatore Academic Hospital of L’Aquila, 67100 L’Aquila, Italy; 3 Unit of Anesthesiology, Intensive Care Medicine, and Pain Medicine, Department of Anesthesia and Critical Care, Department of Medicine, Surgery, and Dentistry, University of Salerno, 84082 Baronissi (Salerno), Italy; 4 Anesthesiology, Critical Care and Pain Medicine Division, Department of Medicine and Surgery, University of Parma, 43121 Parma, Italy; 5 Pediatric Anesthesia and Intensive Care Unit AOU Delle Marche, Salesi Children’s Hospital, 60121 Ancona, Italy; 6 Department of Anesthesiology, Intensive Care and Pain Treatment, University of L’Aquila, 67100 L’Aquila, Italy; 7 Department of Anesthesia and Intensive Care, Hospital of Santa Croce Di Moncalieri, 10024 Turin, Italy; 8 Department of Anesthesia and Intensive Care, Spedali Civili Di Brescia, 25121 Brescia, Italy; 9 Department of Psychology, University of Campania Luigi Vanvitelli, 8100 Caserta, Italy

**Keywords:** Burnout, Maladaptive personality traits, Anesthesiology, Emotional exhaustion, Depersonalization, Personal accomplishment, Maslach Burnout Inventory, Critical care

## Abstract

**Background:**

Burnout is a maladaptive response to chronic stress, particularly prevalent among clinicians. Anesthesiologists are at risk of burnout, but the role of maladaptive traits in their vulnerability to burnout remains understudied.

**Methods:**

A secondary analysis was performed on data from the Italian Association of Hospital Anesthesiologists, Pain Medicine Specialists, Critical Care, and Emergency (AAROI-EMAC) physicians. The survey included demographic data, burnout assessment using the Maslach Burnout Inventory (MBI) and subscales (emotional exhaustion, MBI-EE; depersonalization, MBI-DP; personal accomplishment, MBI-PA), and evaluation of personality disorders (PDs) based on DSM-IV (*Diagnostic and Statistical Manual of Mental Disorders Fourth Edition*) criteria using the assessment of DSM-IV PDs (ADP-IV). We investigated the aggregated scores of maladaptive personality traits as predictor variables of burnout. Subsequently, the components of personality traits were individually assessed.

**Results:**

Out of 310 respondents, 300 (96.77%) provided complete information. The maladaptive personality traits global score was associated with the MBI-EE and MBI-DP components. There was a significant negative correlation with the MBI-PA component. Significant positive correlations were found between the MBI-EE subscale and the paranoid (*r* = 0.42), borderline (*r* = 0.39), and dependent (*r* = 0.39) maladaptive personality traits. MBI-DP was significantly associated with the passive-aggressive (*r* = 0.35), borderline (*r* = 0.33), and avoidant (*r* = 0.32) traits. Moreover, MBI-PA was negatively associated with dependent (*r* =  − 0.26) and avoidant (*r* =  − 0.25) maladaptive personality features.

**Conclusions:**

There is a significant association between different maladaptive personality traits and the risk of experiencing burnout among anesthesiologists. This underscores the importance of understanding and addressing personality traits in healthcare professionals to promote their well-being and prevent this serious emotional, mental, and physical exhaustion state.

**Supplementary Information:**

The online version contains supplementary material available at 10.1186/s44158-024-00171-5.

## Background

Burnout (BO) is defined as a maladaptive response to chronic stress [1]. As widely demonstrated, helping professionals, such as clinicians, are at the greatest risk for this emotional, mental, and physical exhaustion state [2–5].

Moreover, there is evidence in the literature about the risk of anesthesiologists regarding burnout, although with different connotations based on the national context [6–9].

Maladaptive personality traits, such as paranoid (pervasive and unwarranted mistrust and suspiciousness of others), are defined as enduring, inflexible traits that deviate significantly from cultural expectations, impairing an individual’s social, occupational, or personal functioning [10–12]. These traits, while present in various degrees among individuals, become particularly problematic when their expression is rigid and dysfunctional, contributing to significant distress or impairment [13–16]. Personality disorders, as categorized in the *Diagnostic and Statistical Manual of Mental Disorders Fourth Edition* (DSM-IV) [17], represent the extreme manifestations of these maladaptive traits, characterized by pervasive and chronic patterns of behavior and inner experience that are deeply ingrained and lead to functional impairments or subjective distress. The continuum between maladaptive personality traits and personality disorders is recognized in recent psychopathological research, which supports a dimensional rather than a categorical approach to personality assessment. This perspective suggests that personality disorders can be seen as quantitatively extreme variants of commonly shared personality dimensions [18–20]. The relationship between maladaptive traits and the DSM criteria for personality disorders is crucial as it highlights how these traits align with broader personality dimensions assessed by the DSM-5’s Alternative Model for Personality Disorders [21, 22]. This alignment supports the idea that descriptive criteria of personality disorders and maladaptive traits can significantly inform the understanding of personality pathology when considered as part of a dimensional framework [23, 24].

Notably, pieces of evidence suggested an interplay between some maladaptive traits and the risk of burnout [25]. Van der Wal et al. conducted a study on the burnout phenomenon using the *five-factor model* (FFM), in a sample of Dutch anesthesiologists [26]. This approach is useful for distinguishing five different personality traits associated with work stress, but it is particularly valuable in the field of social psychology. An analysis conducted using clinical tools and more focused on the burnout may offer greater insights into the prevention and treatment of this clinical condition.

Nevertheless, to date, the associations of personality features with burnout in anesthesiologists have not been extensively explored.

In a previous study, we investigated the correlation between burnout, alexithymia, and psychological symptoms [27]. In this article, we provide a more in-depth analysis of the topic.

In this secondary analysis, to evaluate the specific and general dimensions of DSM personality pathology that reflect maladaptive degrees of functioning, we used a consolidated measure [28].

As will be specified below, this tool provides a structured and reliable method for quantifying personality dysfunction, making it valuable for clinical research.

In our research, we examine maladaptive personality traits conceptualized as dimensions that cumulatively indicate specific characteristics and general severity of personality pathology.

Based on these assumptions, we aim to highlight how this dimensional assessment of maladaptive personality functioning can improve our understanding of the role in the etiology and progression of burnout among medical professionals.

Dimensions of maladaptive and pathological personality functioning may play a key role in influencing characteristic aspects of burnout. More specifically, burnout is characterized by emotional exhaustion, depersonalization or cynicism, and a reduced sense of personal accomplishment. These key characteristics of burnout share some overlap with common symptoms and interpersonal difficulties found in several personality disorders. For example, emotional exhaustion and detachment in burnout are analogous to the limited emotional experience and withdrawal found in schizoid and avoidant personality disorders. The cynical and callous attitude toward others in burnout resonates with the antagonistic and exploitative patterns observed in narcissistic, antisocial, and paranoid personality disorders. Furthermore, the reduced sense of efficacy and poor outcomes in burnout align with feelings of inadequacy, negative self-perceptions, and pervasive ineptitude that characterize personality pathology. Given these connections, it is plausible that the maladaptive personality traits that underlie personality disorders may confer vulnerability to the development of the debilitating psychological syndrome of burnout over time when confronted with chronic occupational stress.

## Methods

The research was granted ethical approval by the Ethics Committee of L'Aquila and Teramo under protocol number 0024436/20 (Chairman Dr. Goffredo Del Rosso). All participants willingly volunteered after receiving a thorough explanation of the study, and written informed consent was obtained from all the subjects, and their involvement adhered to the “Ethical Principles of Psychologists and Code of Conduct.” All methods were performed in accordance with the ethical standards as laid down in the Declaration of Helsinki and its later amendments or comparable ethical standards.

This manuscript adheres to the applicable Strengthening the Reporting of Observational Studies in Epidemiology (STROBE) guidelines (www.strobe-statement.org).

### Participants

For this secondary analysis, we implemented the results of a previously described survey on physicians of the AAROI-EMAC association (Italian Association of Hospital Anesthesiologists, Pain Medicine Specialists, Critical Care, and Emergency Physicians) [27]. Data were collected during refresher courses at the AAROI-EMAC Simulation Center, SimuLearn®, in February 2020.

To maintain the integrity of the study, individuals with a history of psychiatric diagnoses and/or substance-related disorders, as well as those with a history of psychotropic drug use, were excluded from participation.

### Instruments

These measurements were part of a comprehensive set of questionnaires spanning the fields of psychology, medicine, and clinical risk management. The survey encompassed seven sections: (1) demographic information, (2) turnover intent, (3) personality, (4) burnout, (5) work engagement, (6) work context, and (7) job satisfaction. The sociodemographic questionnaire can be found in the supplementary material of the article.

The questionnaire was administered in “pencil-and-paper” mode to participants in the AAROI-EMAC refresher courses at the SimuLearn® Simulation Center in Bologna, Italy, in February 2020. The choice of a “pencil-and-paper” questionnaire was based on the need to have a high number of complete responses, given the amount of items to be administered. The sociodemographic questionnaire includes questions to which it was possible to answer “yes” or “no” or with multiple options and some questions in which participants were asked to express an opinion according to a 5-item Likert scale.

#### Maslach Burnout Inventory survey

The Maslach Burnout Inventory (MBI) comprises 22 questions, which are categorized into three subscales or dimensions [29]. Emotional exhaustion (MBI-EE) pertains to feeling emotionally drained due to work-related factors. It is assessed through nine items (e.g., “I feel emotionally drained from my work”). Depersonalization (MBI-DP) involves exhibiting an impassive and impersonal response toward those under one’s care, along with a loss of positive attitudes towards oneself, others, and the world. This dimension is evaluated using five items (e.g., “I feel I treat some friends as if they were impersonal objects”). Finally, personal accomplishment (MBI-PA) encompasses reduced personal competence, feelings of frustration, anger, loss of self-esteem, a desire to change jobs, and a lack of success in one’s work. It is measured through eight items (e.g., “I feel I’m positively influencing other people’s lives through my work”) [29].

Each item is rated on a 7-point Likert-type scale, ranging from 0 (never) to 6 (every day). The potential score ranges from 0 to 54 for MBI-EE (high risk: > 26; moderate-high risk: 17–26; moderate or less: < 17), 0 to 30 for MBI-DP (high risk: > 12; moderate-high risk: 7–12; moderate or less: < 7), and 0 to 48 for MBI-PA (high risk: < 32; moderate-high risk: 32–38; moderate or less: > 38) [30]. Elevated burnout levels manifest as high scores on MBI-EE and MBI-DP, along with low scores on MBI-PA. In our study, we employed the validated Italian-language version of the tool [29].

### Assessment of personality severity and maladaptive dimensions

To measure dimensional PD trait scores, we used the Assessment of DSM-IV Personality Disorders Questionnaire (ADP-IV) [28]. The ADP-IV design allows a dimensional trait score and a categorical PD diagnosis for each of the DSM-IV PDs. The ADP-IV is a paper–pencil self-report instrument consisting of 94 items representing the 80 criteria of the 10 DSM-IV PDs and the 14 research criteria of the depressive and the passive-aggressive PDs. Each trait question is rated on a 7-point Likert scale. Internal consistency of the ADP-IV dimensional PD scales is good [28, 31], and test–retest reliability and concurrent validity of the dimensional ADP-IV trait scores are also satisfactory [28, 31]. Most importantly, the ADP-IV showed good concordance with the SCID-II interview [31] and may therefore be considered an economical and valid alternative to semi-structured interviews. In keeping with Hopwood et al. [14], we provided a general personality dysfunction score by adding all DSM-IV trait scores. This approach is consistent with the notion that the sum score of all PD criteria captures the very nature of global and unspecific personality impairment, that is, the variance shared among PD dimensions.

We implemented the severity measure as the sum of all the ADP-IV dimensional trait scores criteria reported by the whole sample [28]. Therefore, personality disorder severity score (PDSEV) represented the general dimensions of PD symptoms severity in the sample. Previous studies revealed acceptable psychometric properties of the ADP-IV [28, 29]. With respect to internal consistencies, we observed Cronbach’s alpha values ranging between 0.60 and 0.85 in the whole study sample.

### Statistical analysis

In particular, to determine how general and specific personality traits influence burnout and its components (emotional exhaustion, depersonalization, and reduced personal accomplishment), we performed a series of independent linear regression analyses. While some correlation among the predictors was anticipated, it was essential to uncover the unique associations between maladaptive personality dimensions and the various facets of burnout. Specifically, we conducted multiple independent linear regression analyses to predict the mean scores of burnout dimensions using personality trait scores, thereby illuminating the distinctiveness of these relationships.

The significance level for hypothesis testing was initially set at a *p*-value of 0.05. Given the number of comparisons conducted, particularly in the analyses corresponding to Tables 2 and 3, there is an inherent risk of type I errors due to multiple statistical testing. To mitigate this risk, we have applied the Bonferroni correction to adjust the significance levels considering at least 12 comparisons.

This adjustment is crucial in ensuring that the likelihood of false positives is minimized. Under the Bonferroni correction, the significance level is divided by the number of comparisons made, thus setting a more stringent criterion for determining statistical significance.

All analyses were controlled for significant demographic characteristics including age and gender, and missing data were reported. Coefficients were standardized to facilitate interpretation, and the analyses were conducted using SPSS 20 (IBM, 2017).

## Results

From 310 respondents, 300 physicians (96.77%) provided complete information for the variables used in this study. As we described in a previous paper [27], of the 300 participants, 101 were male and 199 female; 117 participants were aged between 25 and 39 years, and 183 participants were aged between 40 and 70 years, with an average age of 44.05 years and STD ± 9.51. Regarding the type of structure in which the participants worked: 9 in a private hospital; 214 in a public hospital; 21 in an IRCCS (Istituto di Ricerca e Cura a Carattere Scientifico); 23 in a university; 27 in a mixed hospital (public and private); 5 in ambulance and 1 missing result.

Regarding the job setting, 163 participants worked in the operating room, 107 in intensive care units, 13 in palliative care/pain unit, 7 in other services, and 10 missing.

As we described in a previous article [27], 88 (29,3%) participants were at high risk for emotional exhaustion and 109 (36.3%) at moderate risk, 56 (18.7%) participants are at high risk for depersonalization and 87 (29%) at moderate risk, and 94 (31.3%) participants are at high risk for personal accomplishment and 102 (34%) at moderate risk.

Personality dimensions and MBI subscales were mostly significantly correlated. With respect to the maladaptive personality traits global score (i.e., PD severity), the MBI results were strongly associated with the MBI-EE component (*r* = 0.44) and less with the MBI-DP subscale. Furthermore, there was a weak but significant negative correlation with the MBI-PA component.

The MBI-EE score had the highest significant positive correlations with the paranoid (*r* = 0.42), borderline (*r* = 0.39), and dependent (*r* = 0.39) maladaptive personality traits.

The score of MBI-DP was significantly associated with the passive-aggressive (*r* = 0.35), borderline (*r* = 0.33), and avoidant (*r* = 0.32) traits. Moreover, MBI-PA was best significantly negatively associated with dependent (*r* =  − 0.26) and avoidant (*r* =  − 0.25) maladaptive personality features. Result correlations between MBI sub-scales and ADP-IV personality trait scores are reported in Table 1.

**Table 1:** Correlations between MBI *sub-scales and ADP-IV Personality Trait Scores (n* = *300)*

	MBI emotional exhaustion (MBI-EE)	MBI depersonalization (MBI-DP)	MBI personal accomplishment (MBI-PA)
PD Global Score	0.440**	0.332**	− 0.196**
PAR	0.420**	0.318**	− 0.130*
SZ	0.272**	0.175**	− 0.178**
ST	0.353**	0.221**	− 0.156**
AS	0.237**	0.259**	− 0.095
BDL	0.391**	0.333**	− 0.162**
HIS	0.349**	0.272**	− 0.101
NAR	0.314**	0.245**	0-.058
AV	0.353**	0.320**	− 0.254**
DEP	0.392**	0.310**	− 0.269**
O-C	0.344**	0.097	− 0.046
NOS-DE	0.350**	0.288**	− 0.207**
NOS-PA	0.359**	0.352**	− 0.201**

Criterion: Maslach Burnout Inventory, emotional exhaustion, depersonalization, personal accomplishment. ADP-IV Assessment of DSM-IV Personality Disorders Trait Scores: *PAR* Paranoid, *SZ* Schizoid, *ST* Schizotypal, *AS* Antisocial, *BDL* Borderline, *HIS* Histrionic, *NAR* Narcissistic, *AV* Avoidant, *DEP* Dependent, *O-C* Obsessive–compulsive, *NOS-DE* Depressive, *NOS-PA* Passive-aggressive

^*^p < 0.05

^**^p < 0.001

Subsequently, multiple regression models were designed to explore whether maladaptive personality traits independently accounted for variances in burnout component scores, and results are reported in Table 2.

**Table 2:** Prediction of MBI sub-scales in ADP-IV Personality Trait Scores (*n* = 300)

	MBI emotional exhaustion (MBI-EE) *Std. β (SE); p*	MBI depersonalization (MBI-DE) *Std. β (SE); p*	MBI personal accomplishment (MBI-PA) *Std. β (SE); p*	Model parameters R^2^; F; p MBI-EE^a^; MBI-DE^b^; MBI-PA^c^
Predictor				
PD Global Score	0.44** (0.10); 0.00	0.33** (0.00); 0.00	− 0.19** (0.00); 0.00	^a^0.19; 69.57; 0.000 ^b^0.11; 35.93; 0.000 ^c^0.38; 11.54; 0.001
PAR	0.23* (0.14), 0.006	0.19* (0.08), 0.022	0.07 (0.10) n.s	^a^0.22; 6.74; 0.000
SZ	− 0.00 (0.14) n.s	− 0.03 (0.08) n.s	− 0.10 (0.10) n.s	^b^0.23; 7.28; 0.000
ST	0.07(0.16) n.s	− 0.15 (0.09) n.s	− 0.05 (0.12) n.s	^c^0.14; 4.00; 0.00
AS	− 0.05 (0.24) n.s	0.07 (0.14) n.s	0.06 (0.18) n.s	
BDL	0.09 (0.15) n.s	0.06 (0.08) n.s	− 0.03 (0.11) n.s	
HIS	0.03 (0.20) n.s	0.04 (0.11) n.s	0.07 (0.15) n.s	
NAR	− 0.09 (0.15) n.s	− 0.00 (0.09) n.s	0.13 (0.11) n.s	
AV	0.03 (0.15) n.s	0.18* (0.08) 0.035	− 0.14 (0.11) n.s	
DEP	0.16 (0.15) n.s	0.11 (0.09) n.s	− 0.32*** (0.11) 0.001	
O-C	0.04 (0.13) n.s	− 0.39*** (0.07) 0.00	0.25*** (0.09) 0.005	
NOS-DE	− 0.01 (0.14) n.s	0.07 (0.08) n.s	0.02 (0.10) n.s	
NOS-PA	0.02 (0.18) n.s	0.24** (0.10) 0.01	− 0.20* (0.13) 0.039	

Criterion: Maslach Burnout Inventory, emotional exhaustion, depersonalization, personal accomplishment. Predictors: DP-IV Assessment of DSM-IV Personality Disorders Trait Scores: *PAR* Paranoid, *SZ* Schizoid, *ST* Schizotypal, *AS* Antisocial, *BDL* Borderline, *HIS* Histrionic, *NAR* Narcissistic, *AV* Avoidant, *DEP* Dependent, *O-C *Obsessive–compulsive, *NOS-DE* Depressive, *NOS-PA* Passive-aggressive

^*^p < 0.05

^**^p < 0.01

^***^p < 0.001

The personality global severity score was significantly associated with all burnout components. The overall score predicted MBI-EE and MBI-DE scores (*β* = 0.44 and *β* = 0.33, respectively). A negatively significant association with MBI-PA (*β* =  − 0.19) was calculated.

With respect to maladaptive personality trait scores, the paranoid trait was the unique significant predictor of the MBI-EE component (*β* = 0.33). The MBI-DE component was significantly negatively predicted, with a standard significance level set at alpha = 0.05, by obsessive–compulsive trait (*β* =  − 0.39) and positively predicted by passive-aggressive trait (*β* = 0.24), paranoid trait (*β* = 0.19), and avoidant trait (*β* = 0.18). However, considering the Bonferroni correction factor which brings the threshold value at alpha = 0.004, it becomes appropriate to consider obsessive–compulsive trait (*β* =  − 0.39) the best significant predictor.

Finally, the MBI-PA component was, with alpha = 0.05, significantly negatively best predicted by the dependent trait (*β* =  − 0.32) and positively predicted by the obsessive–compulsive trait (*β* = 0.23), with a negative significant association with the passive-aggressive trait (*β* =  − 0.20). Similarly to the previous consideration, considering alpha = 0.004, the MBI-PA component was negatively significantly predicted only by the dependent personality dimension (*β* =  − 0.32).

The results of the multiple regression models are presented in Table 2.

We investigated the potential associations between environmental conditions at risk of burnout and maladaptive traits, and significative results are reported in Table 3. It should be noted that subjects affected by deprivation from sunlight in the operating room have a greater association with maladaptive traits (PD severity: low mean 176.09, high mean 188.61 F(1, 286) = 3.79, *p* = 0.050). Similarly, anesthesiologists who feel uncomfortable wearing a uniform have a higher average mean score in PD severity (low mean 179.32, high mean 201.40 F(1, 290) = 6.50, *p* = 0.011).

**Table 3:** Participants’ characteristics and total mean scores of burnout and PD Severity Global Score

	Variables	f (f%)	MBI Emotional exhaustion (EE) Mean ± SD (F; p)	MBI Depersonalization (DE) Mean ± SD (F; p)	MBI Personal accomplishment (PA) Mean ± SD (F; p)	PDSEV Global Score (PDSEV) Mean ± SD (F; p)
Sex *n* = 300	Male	101 (33.7)	19.21 ± 9.99	7.68 ± 6.10	35.54 ± 7.19	181.86 ± 54.87
	Female	199 (66.3)	22.17 ± 10.2 (5.65; 0.018*)	6.71 ± 5.72 (1.82; n.s.)	34.24 ± 7.35 (2.11; n.s.)	183.17 ± 53.52 (0.38; n.s.)
Age *n* = 300	25–39	117 (39.0)	20.17 ± 9.63	8.50 ± 6.03	34.48 ± 6.85	188.57 ± 55.26
	40–70	183 (64.0) (0.177, n.s.)	21.81 ± 10.6 (12.30; 0.001*)	6.10 ± 5.61 (0.137; n.s.)	34.80 ± 7.61 (0.44; n.s.)	179.03 ± 52.83 (2.178; n.s.)
Have you ever had the feeling of being mobbed? *n* = 300	No	167 (55.6)	19.01 ± 10.37	6.67 ± 5.49	35.04 ± 7.30	173.33 ± 50.22
	Yes	133 (44.33)	23.90 ± 9.46 (17.77; 0.000**)	7.49 ± 6.34 (1.14; n.s.)	34.23 ± 7.33 (0.95; n.s.)	194.76 ± 56.19 (11.78; 0.001*)
Have you a conflictual relationship with surgeons? *n* = 298	Low	182 (61.07)	19.62 ± 10.31	5.79 ± 5.53	35.31 ± 7.56	175.71 ± 53.11
	High	116 (38.92)	23.62 ± 9.79 (11.05, 0.001*)	8.89 ± 5.77 (21.58; 0.000**)	33.65 ± 6.86 (3.65; n.s.)	194.60 ± 53.38 (8.69; 0.003*)
Have you a conflictual relationship with other anesthesiologists? *n* = 300	Low	126 (42.00)	19.53 ± 10.45	5.65 ± 5.00	35.12 ± 7.44	170.83 ± 50.76
	High	176 (56.00)	22.37 ± 9.97 (5.69, 0.018*)	8.04 ± 6.27 (12.41; 0.000**)	34.36 ± 7.22 (0.79; n.s.)	191.26 ± 54.60 (10.54; 0.001*)
Is it hard for you to explain your work to patients? *n* = 300	Low	159 (53.00)	18.11 ± 9.57	5.42 ± 5.12	36.07 ± 7.31	171.93 ± 47.92
	High	141 (47.00)	24.63 ± 9.93 (33.37; 0.000**)	8.86 ± 6.17 (27.85; 0.000**)	33.11 ± 7.02 (12.73; 0.000**)	195.12 ± 57.75 (14.05; 0.000**)
Have you ever benefited from psychotherapeutic? *n* = 300	No	233 (77.66)	20.02 ± 9.77	6.61 ± 5.65	34.68 ± 7.27	171.17 ± 52.01
	Yes	67 (22.33)	25.19 ± 10.94 (13.77; 0.000**)	8.50 ± 6.47 (5.43; 0.020*)	34.67 ± 7.49 (0.00; 0.020*)	187.53.18 (6.25; 0.013*)

Legend: *p < 0.05; **p < 0.001

## Discussion

Focusing on the association between maladaptive personality dimensions and burnout risk among anesthesiologists, our results found a significant association with both the general severity of personality and unique traits.

The study has identified certain personality traits that are particularly susceptible to being associated with burnout among our anesthesiologists’ sample. These traits include paranoid, dependency, avoidance, passive-aggressive, and obsessive–compulsive. However, it is essential to note that none of these traits individually exposes anesthesiologists to the risk of experiencing all three dimensions of burnout, namely MBI-EE, MBI-DP, and reduced MBI-PA. The results indicate that the paranoid trait is the most significant predictor of MBI-EE, suggesting that individuals with higher levels of paranoid ideation may be more prone to emotional exhaustion in the workplace. On the other hand, the obsessive–compulsive personality trait exhibits the highest predictive capacity for both depersonalization (MBI-DP) and reduced personal accomplishment (MBI-PA). This implies that individuals with obsessive–compulsive traits may be at a higher risk of experiencing depersonalization and a decreased sense of personal accomplishment in their professional roles.

Interestingly, the dependent, avoidant, and passive-aggressive traits are also noteworthy predictors, but they impact MBI-DP and MBI-PA in contrasting ways. For instance, the dependent trait naturally leads to lower MBI-PA due to its nature of relying on others, but it does not necessarily result in emotional exhaustion or a lack of empathy. Similarly, the passive-aggressive trait inherently exhibits depersonalized behavior due to low self-esteem and reduced personal accomplishment. However, this depersonalized behavior does not necessarily lead to emotional breakdown.

While the Bonferroni correction imposes a more stringent criterion for determining statistical significance, it is important to note that this adjustment is a conservative approach to control for the increased likelihood of type I errors due to multiple comparisons. Despite the formal requirements imposed by the Bonferroni correction, the identified significant associations still provide valuable insights into the relationships among the variables under investigation. Although some associations may no longer reach the corrected significance threshold, they can still be considered noteworthy and relevant to the objectives of the study. Particularly, the associations that exhibit stronger effect sizes or align with theoretical expectations or previous empirical findings merit further consideration and interpretation. The Bonferroni correction serves as a safeguard against false positives, but it should not overshadow the substantive implications of the observed relationships, especially when they are consistent with the broader context of the research area.

### Study limitations

As this study is based on a secondary analysis of a preexisting dataset, it is not based on a specific hypothesis. The associations found should be considered exploratory, as the cross-sectional nature of the study limits causal inferences. Consequently, the findings are best understood in the context of associations between personality traits and the prevalence of burnout rather than predicting the risk of developing it. Future longitudinal research is warranted to more accurately establish the temporal relationship between these traits and burnout.

However, the results should be considered in the light of different limitations. Theoretical models in psychology often emphasize the role of maladaptive traits in the development of psychological and emotional distress, including burnout [32, 33]. Maladaptive traits are believed to be more directly related to negative outcomes, making them a relevant area of study in the context of burnout [34, 35]. Moreover, identifying and understanding the role of maladaptive traits in burnout among anesthesiologists can have practical implications for interventions and preventive strategies [36].

On the other hand, personality is a multifaceted construct, and both adaptive and maladaptive traits can interact and influence an individual’s well-being. Future research could indeed consider a more comprehensive approach by examining a wider range of personality traits to gain a more holistic understanding of their impact on burnout and well-being in this category of healthcare professionals.

Another limitation of the study concerns the limited generalizability of its findings. While the study included a large sample of Italian anesthesiologists, the study’s findings are primarily applicable to this particular population [37].

Finally, the study exclusively relied on self-report measures for all evaluations. This approach is susceptible to limitations based on individuals’ self-awareness when completing the measures [28, 29]. To enhance our understanding of the connection between personality traits and various facets of psychopathology concerning burnout, future research should consider employing research strategies that incorporate both self-reports and external assessments.

### Study perspectives and future directions

Based on the insights gained from this study, several promising avenues for future research can further advance our understanding of the interplay between maladaptive personality traits and burnout among anesthesiologists. Conducting longitudinal studies, for example, can provide a dynamic perspective on how maladaptive personality traits and burnout evolve over time. Examining these changes within the same cohort of healthcare professionals can help identify critical periods for intervention and resilience building. Consequently, this approach includes assessing the long-term effectiveness of interventions and their impact on job satisfaction, patient care, and overall well-being.

Moreover, given the identified associations, researchers can develop targeted interventions aimed at mitigating burnout risks among anesthesiologists with specific maladaptive traits.

Some examples of possible interventions are as follows:

- Measuring burnout levels of anesthesiologists and intensivists periodically by the occupational medicine service in collaboration with a team of psychologists
- Following the measurements of burnout levels, planning interventions within the anesthesia unit to redistribute workloads, make tasks more varied, and resolve conflicts between colleagues
- Implementing relaxation techniques, such as mindfulness, and establishing a psychological support program (through voluntary interviews) with a team of psychologists, also based on personality traits: increase coping strategies and resilience capacity
- Reducing the number of consecutive hours of work ensuring adequate rest
- Rethinking the career system, ensuring horizontal progression with increasing responsibilities, based on areas of expertise with particular reference to new technological developments in anesthesia, critical care, and pain medicine [38–40]
- Reducing medical-legal litigation which is a source of work-related stress
- Training anesthesia chiefs to manage conflicts within the team and to manage, if not actually enhance, the difficult human resources

AAROI-EMAC is engaged in translating the results of the study into healthcare policies, from a translational medicine perspective, for example, by organizing managerial courses for anesthesia chiefs, investing in reducing medical-legal litigation and organizing courses on subspecialty topics, which have a high impact on an emotional level [41–43].

Expanding the scope of research to include an international or multicultural perspective can yield valuable insights into how the interplay between personality traits and burnout varies across different healthcare systems and cultural contexts. Remarkably, comparative analysis can inform the development of culturally sensitive interventions. It also involves collaborative efforts with other medical specialties and healthcare professionals to provide a holistic view of burnout in the medical field. In this context, comparative studies can highlight unique challenges faced by anesthesiologists and lead to tailored solutions.

Our results suggest that further investigation into the impact of workplace and environmental factors on the development of maladaptive traits and burnout is warranted. Understanding how organizational policies, workloads, and team dynamics contribute to these dynamics can guide healthcare institutions in creating more supportive and nurturing work environments. Furthermore, exploring the resilience and coping strategies employed by anesthesiologists with maladaptive traits can offer insights into effective ways of mitigating burnout risks. In other words, identifying adaptive strategies can inform interventions and training programs.

## Conclusions

Our work has highlighted the significant associations that exist between maladaptive personality dimensions and burnout in anesthesiologists. Although this exploratory study does not allow for direct inferences regarding the risk of burnout due to its cross-sectional design, the emerging data provide valuable insights that should inform future research. Such research could focus on understanding these dimensions in greater detail and identifying potential vulnerability factors. These factors may serve as specific intervention targets in healthcare personnel, particularly in the class of individuals we studied. In our study, maladaptive traits such as paranoid, dependent, avoidant, passive-aggressive, and obsessive–compulsive tendencies were linked to high scores of burnout. Recognizing these associations is critical to informing tailored interventions and support mechanisms that address the unique traits of this category of healthcare professionals. By identifying and addressing these traits early, healthcare organizations can cultivate a supportive environment that prioritizes well-being and resilience, thus better equipping their workforce to handle the challenges associated with burnout.

### Supplementary Information

Additional file 1: Appendix 1. Domande sociodemografiche

## Data Availability

The datasets used and/or analysed during the current study are available from the corresponding author on reasonable request.

## Abbreviations

BO: Burnout
AAROI-EMAC: Italian Association of Hospital Anesthesiologists, Pain Medicine Specialists, Critical Care, and Emergency Physicians
AS: Antisocial
AV: Avoidant
BDL: Borderline
DEP: Dependent
DSM-IV: *Diagnostic and Statistical Manual of Mental Disorders Fourth Edition*
FFM: Five-factor model
HIS: Histrionic
IRCCS: Istituto di Ricerca e Cura a Carattere Scientifico
MBI: Maslach Burnout Inventory
MBI-DP: Depersonalization
MBI-EE: Emotional exhaustion
MBI-PA: Personal accomplishment
NAR: Narcissistic
NOS-DE: Depressive
NOS-PA: Passive-aggressive
O-C: Obsessive-compulsive
PA: Paranoid
PD: Personality disorders
PDSEV: Personality disorder severity score
SD: Standard deviation
ST: Schizotypal
SZ: Schizoid
