# Supplementary material for: Maladaptive personality traits are associated with burnout risk in Italian anesthesiologists and intensivists: a secondary analysis from a cross-sectional study
Source: J Anesth Analg Crit Care. 2024 Jun 21;4:36. doi: 10.1186/s44158-024-00171-5 (PMC11193186; doi:10.1186/s44158-024-00171-5)
Supplement: Supplementary file 1 — Additional file 1: Appendix 1. Domande sociodemografiche [file 44158_2024_171_MOESM1_ESM.docx]

**Appendice 1. Domande sociodemografiche**

1 Did you study medicine in your city? YES NO [ ]

2 If you have not studied medicine in your city you have lived: (1) since

friends; (2) from relatives; (3) with other students; (4) alone; (5) in a

Institute

3 How many years did it take you to earn your degree?. _________ .

4 Did you study at a public university? YES NO [ ]

5 How much influence did your parents have on your choice to study?

medicine? (1) a lot; (2) little; (3) not at all; (4) were against (1) (2) (3) (4)

6 If you could go back, would you still enroll in medicine? YES NO [ ]

7 You chose the specialization in anesthesia and resuscitation or you wanted to

do anything else? YES [ ] NO [ ] What?_____________

8 You specialized in anesthesia and resuscitation after have you achieved any other specializations? If so which ones? YES NO [ ] Spec. ________________________

9 Did you graduate from the same university where you graduated? YES NO [ ]

10 Do you work in your home city? YES NO [ ]

11 Do you work in the same city where you specialized? YES NO [ ]

12 What type of hospital do you work in? Select the main item (1) private hospital; (2) public hospital; (3) private hospital

Accredited; (4) IRCCS; (5) university hospital; (6) ambulance (1 2 3 4 5 6)

13 The venue where you work is located: (1) North; (2) Center; (3)

South; (4) Islands (1 2 3 4)

14 How many beds does the facility you work at have? Number or none (zero, e.g. 118)

( ____ )

15 Do you work in the same venue you attended as a trainee? YES NO [ ]

16 What type of contract do you have? (1) freelancer; (2)

determined time; (3) indefinite period (1) (2) (3)

17 If you are precarious, how many years have you been in this situation? YES [ ] Years ________ NO [ ]

18 Do you mainly work in which area? (1) anesthesia; (2) intensive care unit-ED; (3) ambulance; (4) cardiac anesthesia; (5) pain relief - palliative care

19 If you work in the operating room (No = 0) what is the principal working field? (1) pediatric, (2) general surgery, (3) ENT, (4) obstetrics, (5) emergency surgery, (6) orthopedics, (7) neurosurgery,

(8) thoracovascular, (9) transplant surgery (0) (1) (2) (3) (4) (5) (6) (7) (8) (9)

20 If you work in the ambulance, do you also do an air ambulance? 118 - YES [ ] NO [ ] ; ELI - YES [ ] NO [ ]

21 If you work in cardiac anesthesia (No = 0) what is your principal field of work? (1) pediatric, (2) adult (0) (1) (2)

22 If you work in pain therapy-palliative care (No = 0) what is your main scope? (1) pediatric, (2) adult (0) (1) (2)

23 At the end of your specialization you would have liked to work in the field in which you are currently employed? YES NO [ ]

24 Do you think the salary you currently receive is adequate? YES NO [ ]

25 Have you ever had disciplinary proceedings from your employer work? YES NO [ ]

26 How scared are you of being sued? Not at all (1) (2) (3) (4) (5) Very much

27 Are you satisfied with the care activity you carry out? YES NO [ ]

28 Does the work you do allow you to cultivate a hobby? YES NO [ ]

29 Does the job you do allow you to practice a sport? YES NO [ ]

30 Have you ever felt mobbed? If yes, from a superior or a colleague of yours? YES NO [ ]

Superior [ ] Colleague[ ]

31 Do you feel satisfied with your career? YES NO [ ]

32 Are you satisfied with the research activity you carry out? YES NO [ ]

33 Are you satisfied with your sleep/wake cycle? YES NO [ ]

34 Do you use benzodiazepines to help you sleep? YES NO [ ]

35 Are you a shift worker?If so, do you think the shift load is too heavy? YES NO [ ] [ ]

YES NO[ ] [ ]

36 Is there a break room in your work environment? If so, does it seem appropriate to you?

YES NO[ ] [ ] YES NO[ ] [ ]

37 Do you miss the doctor-patient relationship? YES NO [ ]

38 Do you have a conflicting relationship with your surgeon colleagues? Not at all (1) (2) (3) (4) (5) Very much

39 Is there a conflictual relationship between colleagues within your service? Not at all (1) (2) (3) (4) (5) Very much

40 If you work in the operating room, does the lack of light bother you? Not at all (1) (2) (3) (4) (5) Very much

41 If you work in uniform, does it bother you not to be able to dress freely? Not at all (1) (2) (3) (4) (5) Very much

42 Do you find it difficult to explain your work to patients? Not at all (1) (2) (3) (4) (5) Very much

43 Does it bother you not to be adequately considered by patients? Not at all (1) (2) (3) (4) (5) Very much

44 Does stress related to the severity of the patients you treat weigh on you? Not at all (1) (2) (3) (4) (5) Very much

45 What weighs on you the most? (1) the death of a patient, (2) the suffering of patients,

(3) long work shifts (1) (2) (3)

46 Do you have a partner? If yes, your partner is one (1) anesthetist; (2) doctor other

spec. (3) other profession? YES NO [ ] (1) (2) (3)

47 The work you do allows you to dedicate yourself as much as you would like to yours

free time? YES NO[ ] [ ]

48 The work you do allows you to dedicate yourself to yours as you would like

affection?

YES NO [ ] [ ]

49 For professional reasons, have you ever received a complaint in the criminal field?

YES NO [ ] [ ]

50 For professional reasons, have you ever received a complaint in the civil field

civil? YES NO [ ] [ ]

51 Have you ever used psychotherapeutic services? If yes, what orientation?­­­­­__________
